# Deep Learning Calculation and Application of Axle Loads in Highway Sensor Data

**DOI:** 10.3390/s24247930

**Published:** 2024-12-11

**Authors:** Lukai Zhang, Xiaoya Wang, Yingping Wang

**Affiliations:** 1 Transport Planning and Research Institute, Ministry of Transport, Chaoyang District, Beijing 100028, China; 18114054@bjtu.edu.cn; 2 Reading Academy, Nanjing University of Information Science and Technology, Pukou District, Nanjing 210044, China; wangxiaoyananjing@163.com

**Keywords:** highway sensors, maintenance management, axle load spectrum, equivalent axle volume, deep learning

## Abstract

Axle load data and traffic survey data are both important outputs of highway sensors. This study targets highways and ordinary national and provincial highways, seeking to calculate axle load spectrum and equivalent axle times across the network. There is often an association in the spatial extent of traffic survey data and axle load detection data in highway networks. Initially, using the Highway Asphalt Pavement Design Specification, it analyzes the demand for these calculations in road sections. Considering the current axle load detection coverage, a method supported by highway traffic data is proposed. For integrating multi-source data, a generalized regression neural network model is established, enabling deep learning calculations. The method is validated and applied to Xuzhou’s highway network. Results show consistency between the calculated average axle load spectrum and actual data. Among validation samples, 3-axle vehicles exhibit the smallest deviation, while 6-axle vehicles show the largest. Calculating equivalent axle numbers reveals the distribution and grading of heavily loaded road sections, aiding maintenance decisions.

## 1. Introduction

The laying of highway sensors has greatly enriched the sources of traffic data. Traffic surveys and axle load detection exhibit an association in the distribution of their monitoring locations, a characteristic that can be leveraged for data imputation. According to the “Highway Asphalt Pavement Design Code” [1,2], the axle load spectrum is defined as the distribution of the number of actions of various axle types within different axle load intervals over a specified road cross-section period. Meanwhile, the equivalent axle volumes are determined by comparing the pressure effect of a single axle to a standard pressure effect of 100 KN, yielding the equivalent axle pressure number for the same road section and time period. With the growth of society and the economy, there has been a significant increase in heavy-haul vehicles, leading to a substantial rise in axle load compression loss on highways. This increase has accentuated the impact on pavement performance. Consequently, in maintenance design and decision-making optimization, the calculation of highway axle load spectrum and equivalent axle volumes has emerged as a focal point of both theoretical research and engineering practice.

Current research on ordinary highway axle loads primarily focuses on two aspects: investigating the causes of subgrade diseases in pavements due to axle loads and examining the stress grade of asphalt pavement degradation under axle load influence. In terms of methodology, large-scale mathematical statistical analysis is predominantly employed to calculate axle load indicators with varying spatial and temporal resolutions.

Among recent significant advancements, Liu et al. [3] employed an analogy model test approach to propose stability criteria for low embankment foundations subjected to excessive axial loads, grounded in critical dynamic stress analysis. Li Sheng et al. [4] conducted three-dimensional solid modeling and numerical simulations using Abaqus finite element software, unveiling a heightened correlation between axle loads exceeding 100 kN and pavement structure depth. Chen Xiang et al. [5] established a three-dimensional finite element model to investigate the axle load spectrum pattern and equivalent axle order interval for hollowed cement concrete pavement slabs. Tao et al. [6] analyzed traffic load characteristics on Togo Highway 1 using dynamic weighing survey data, calculating the axle distribution coefficient for four-axis vehicles. Zhao et al. [7] examined stress responses within the asphalt–soil foundation under varying vehicle operational states and axle loads, deducing the axle load conversion factor based on soil foundation stress analysis. Fang et al. [8] conducted fatigue damage tests utilizing Miner theory and ABAQUS software 1.0, assessing the fatigue damage induced by excessive loads on cement-stabilized macadam subbases. Ling et al. [9] analyzed traffic composition and standard axle load selection, determining the critical load position and axle load conversion factor for concrete slabs through finite element model calculations. Li et al. [10] employed a novel geotechnical grid for asphalt pavement surface reinforcement, conducting field measurements and calculations of the axle load response. Additionally, Li [11] analyzed the deflection of existing road structures under moving loads, comprehensively calculating the stress response.

Yang et al. [12] investigated axle load characteristics of expressways using spectrum data from key points, analyzing patterns in vehicle type, monthly adjustment, and axle load distribution coefficients, and discussed axle overload phenomena. Dai et al. [13] assessed various equivalent axle load conversion methods based on these data for practical applications. Qian et al. [14] proposed a rut equivalent axle load model by analyzing measured expressway data. Wang et al. [15] developed an axle load model from measured data to inform road design and maintenance. Ma et al. [16] compared heavy traffic axle load spectrum parameters with design loads for road design under such conditions. Gao et al. [17] revealed the extent of overload on high-grade roads and suggested preventive measures. Wu et al. [18] reviewed research on axle load impacts on asphalt pavement performance. Wang et al. [19] provided a theoretical foundation for road design and research through a comprehensive review of vehicle load models. Guo et al. [20] emphasized the importance of axle loads in bridge design, including their impact on structures and load conversion methods. Zhao et al. [21] analyzed the current state of axle load management and discussed future trends.

The aforementioned research primarily calculates and analyzes highway axle load spectrum and equivalent axle volumes through experimental means and full samples. However, it inadequately addresses the challenge of predicting and calculating axle load indices in blind areas. Furthermore, the infrastructure for axle load monitoring and statistical collection within our country’s road network lacks widespread coverage. Considering these limitations, data-driven highway axle load calculation methods are becoming increasingly important. This study, taking into account existing data resource conditions, utilizes road traffic observation survey (referred to as “intersection”) data as the primary support. It constructs a generalized regression neural network model, implements a deep learning calculation process, and outputs the entire network’s axle load spectrum, equivalent axle volumes, and other objectives. The Xuzhou highway network serves as an example for verification and application. To improve readability, we have simplified and explained complex technical terms (such as GRNN, axle load, et al.) appearing in the text.

## 2. Definition of Axle Load Spectrum and Equivalent Axle Volumes Calculation

Traffic survey data is primarily obtained through automated traffic survey equipment distributed across the highway network, which records the flow information of different types of vehicles. Axle load data is mainly collected by facilities such as axle load detection stations, highway overload control detection stations, remote law enforcement stations, and bridge health monitoring systems, providing detailed information on vehicle axle loads. This description will help readers better understand the sources and reliability of the data. Combined with the physical implications and actual survey statistical context, this paper elucidates the computational definitions of highway axle load spectrum and equivalent axle volumes.

### 2.1. Definition of Axle Load Spectrum Calculation

The axle load spectrum is defined as the proportion of different axle types from various vehicles within distinct axle load intervals, representing the axle load distribution coefficient for each axle type. Building upon actual investigations and statistical traffic flow data, this paper proposes the calculation definition of the axle load spectrum, as depicted in Equation (1):

(1) $$ALDF_{mij}=ND_{mij}/NA_{mi}$$

where

m: Vehicle-type index variable, denoting different vehicle types based on combinations of equal axles, such as single axle, twin axle, and triple axle.

i: Axis-type index variable, representing single axis, double axis, and triple axis configurations.

j: Axle weight interval index variable, corresponding to 2.5 kN, 4.5 kN, 9.0 kN, and 13.5 kN for single, duplex, and triplex axes, respectively.

$ALDF_{mij}$: Axle load distribution coefficient of axle type i in the axle load section j for vehicle class m within the road segment statistics.

$ND_{mij}$: Number of occurrences of axle type i in section j for vehicle class m within the road segment statistics.

$NA_{mi}$: Total number of occurrences of axle type i for vehicle class m within the road segment statistics.

### 2.2. Definition of Equivalent Axle Volumes Calculation

Equivalent axle volumes signify the frequency at which the load of a single axle is deemed equivalent to a standard axle load. By taking the midpoint value of each axle load interval as the representative axle load for that interval, the equivalent axle volumes for various axle types of different vehicles across different axle load intervals are calculated, as illustrated in Equation (2):

(2) $$EALF_{mij}=c_{1}c_{2}{\left(P_{mij}/P_{s}\right)}^{b}$$

where

$EALF_{mij}$: Equivalent axle number of axle type i in the axle load range j for vehicle class m.

$c_{1}$: Axis group coefficient; when the distance between the front and rear axles exceeds 3 m, it is calculated as a single axis; for shaft spacings less than 3m, the double shaft takes a value of 2.1, and the triple shaft takes 3.2.

$c_{2}$: Wheel group coefficient: 1.0 for the two-wheel group and 4.5 for the single-wheel group.

$P_{s}$: Standard axle load for asphalt pavement, set at 100 kN.

$P_{mij}$: Single axle load (in kN) of axle type i for vehicle class m in the axle load range j, representing the axle load evenly distributed to each single axle for double and triple axles.

b: Conversion index, varying based on the analysis: for asphalt mixture layer fatigue and permanent deformation, b=4; for roadbed permanent deformation, b=5; and for the fatigue of inorganic binder-stabilized layer, b=13.

The coefficients used in axle load calculations are derived from previous research and engineering experience. We have added detailed explanations of these coefficients and provided a review of the relevant literature to strengthen the theoretical foundation. The aforementioned definitions align with the calculation procedure for highway axle loads under actual survey and statistical conditions. Leveraging comprehensive axle load data, the axle load spectrum and equivalent axle volumes for the targeted road sections can be directly computed.

## 3. Deep Learning Method and Process for Highway Axle Load Calculation

Initially, we analyze the principle of data fusion for highway axle load calculation. Subsequently, the structural framework of the generalized regression neural network (GRNN) is delineated.

### 3.1. Intermodulation and Axle Load Data Fusion Principle

The axle load of uniaxial granularity constitutes a pivotal data source, as per the calculation formula and its definition. In the context of highway networks, a limited number of road sections are typically equipped with facilities such as axle load investigation stations, highway overload control detection stations, remote law enforcement stations, and bridge health monitoring systems. Consequently, the availability of axle load data is relatively scarce. Therefore, it becomes imperative to adopt a multi-source data fusion perspective to supplement and align the targeted information. Notably, highway automated traffic survey equipment, which is widely distributed and covers a significant proportion of the network, can serve as a supplementary input data source for comprehensive sampling.

**Figure 1:**
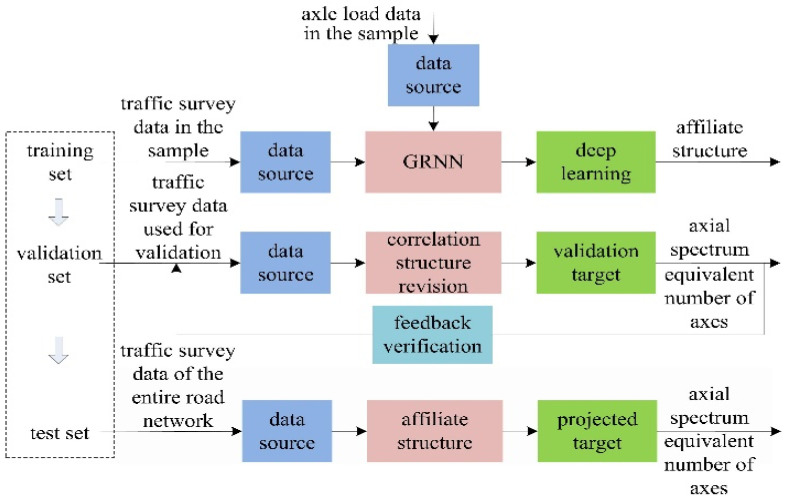
Data fusion calculation principle for highway axle load estimation.

Accordingly, this study proposes a computational process that integrates intermodulation data with axle load detection data. This process involves collecting calculation samples of axle load spectrum and equivalent axle volumes for road sections, conducting deep learning of the associative structure using GRNN, and establishing the input–output relationship. This transfer relationship facilitates the targeted calculation of axle load data blind spots. The principle of data fusion for highway axle load spectrum and equivalent axle volumes is illustrated in Figure 1.

During sample collection, a select few highway sections with axle load detection data are chosen. Axle load spectrum and equivalent axle volumes are computed based on Formulas (1) and (2), respectively. Furthermore, Table 1 presents the analysis of vehicle-type correspondence between intermodulation and axle load data. In the computational implementation, the vehicle type corresponding to the interchange data source is set according to the vehicle type with the target output axle load. We split the dataset into training (70%), validation (15%), and test sets (15%). Random sampling was used to ensure a uniform distribution of vehicle types in each set. Additionally, we employed 5-fold cross-validation to further validate the robustness of the model.

### 3.2. Flow of Generalized Regression Neural Network Structure

**Figure 2:**
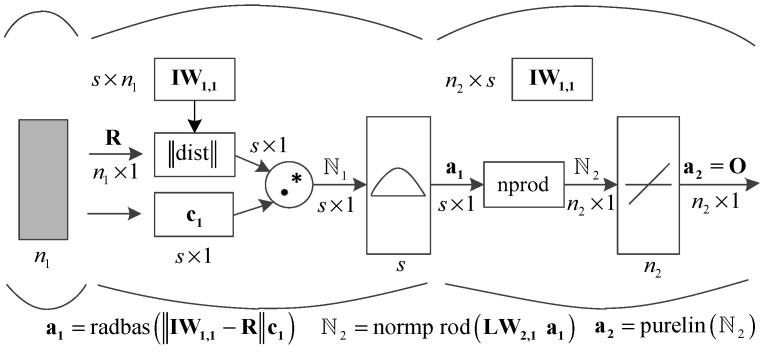
The generalized regression neural network (GRNN) model used in this study.

Based on the proposed data fusion principle for highway axle load calculation, a generalized regression neural network (GRNN) model is constructed to delineate the correlation structure between intermodulation data and axle load data. This model facilitates the computation of highway axle load spectrum and equivalent axle volumes. GRNN is a well-established deep learning framework that, with the aid of data, forms an optimal network structure through sample data training and learning, subsequently enabling the computation of specific target outputs. In the GRNN architecture, input values are initially transmitted to nodes in the intermediate structure layer. Following a transfer function operation, the output values of these nodes are then relayed to nodes in the output layer. Given the inherent complexity of calculating highway axle load spectrum and equivalent axle volumes, a 4-layer GRNN evaluation model is employed. As depicted in Figure 2, this model comprises an input layer, two cascading hidden layers, and an output layer. The number of nodes in both the input and hidden layers is contingent upon the sample size, while the number of nodes in the output layer is determined by the output requirements. Additionally, the four output nodes can be represented as a single normalized multi-element vector.

Within the constructed GRNN model, the analytical expression for the hidden layer weight value is the Euler distance function (denoted as $\left\|\mathrm{dist}\right\|$), which computes the distance between the input layer and the hidden layer weight value $IW_{1,1}$. The hidden layer features an excitation threshold ${\mathbb{N}}_{1}$, and its signal transfer function belongs to the radial basis function (RBF), with the Gaussian function being utilized in implementation. The subsequent network structure is a linear output component, characterized by a normalized dot product weight function (expressed as nprod). The calculation vector ${\mathbb{N}}_{2}$ is derived by dividing the vector elements $a_{1}$ by the sum of the elements $LW_{2,1}$ after performing the dot product of each row of the vector with the weight value matrix. The resultant vector ${\mathbb{N}}_{2}$ is then output through the linear transmission module $a_{2}=\mathrm{purelin}\left({\mathbb{N}}_{2}\right)$.

① The learning structure of GRNNs bears similarities to that of RBF networks, but the numerical forms of the output results differ significantly. The model construction proceeds as follows:

The RBF centers for the hidden layer neurons are determined, establishing a nonlinear mapping R→B, as illustrated in Equation (3).

(3) $$B_{P}=\vec{F}\left(R_{P}\right)\;\;p=1,2,\cdots ,s$$

Guided by the information transmission process of GRNN and supported by foundational data, the mapping ϕ is obtained through the network learning process and approximated via internal network structure learning and training, as depicted in Equation (4).

(4) $$\hat{F}:\;R^{n_{1}}\to R\;\;B=\hat{F}\left(R\right)$$

Let the sample matrix R and output matrix B of the training set be as represented in Equation (5).

(5) $$R=\left[\begin{array}{cccc} r_{1,1} & r_{1,2} & \cdots & r_{1,s}\\ r_{2,1} & r_{2,1} & \cdots & r_{2,s}\\ \vdots & \vdots & & \vdots \\ r_{n1,1} & r_{n1,2} & \cdots & r_{n1,s} \end{array}\right],\;B=\left[\begin{array}{cccc} b_{1,1} & b_{1,2} & \cdots & b_{1,s}\\ b_{2,1} & b_{2,1} & \cdots & b_{2,s}\\ \vdots & \vdots & & \vdots \\ b_{n2,1} & b_{n2,2} & \cdots & b_{n2,s} \end{array}\right]$$

In the formula above, $r_{ij}$ is represented as the input variable i in the training set sample j; $b_{ij}$ denotes the input variable i in the training set sample j; s is the number of samples; $n_{1}$ are the dimensions of input variables; and $n_{2}$ is the dimension of the output variable.

For the hidden layer, the neural network model in this study shares characteristics with the traditional RBF network, where each node corresponds to a single sample s. The RBF center corresponding to each hidden layer node FC is given by Equation (6).

(6) $$FC=R^{\prime }$$

② The excitation threshold for hidden layer nodes is determined. To ensure efficient output in the evaluation calculation, the excitation threshold for each hidden layer node s is defined as shown in Equation (7).

(7) $$c_{1}={\left[c_{11},c_{12},\cdots ,c_{1s}\right]}^{\prime }$$

where $c_{11}=c_{12}=\cdots =c_{1s}=c_{0}/\tau$, $c_{0}$ is the base threshold and τ is the radial basis function of the propagation velocity.

③ The weight value between the hidden layer and the output layer is ascertained. Based on the determined hidden layer nodes and excitation thresholds, the hidden layer node output is computed, as detailed in Equation (8).

(8) $$a_{i}=\exp\left(-{\left\|FC-r_{i}\right\|}^{2}c_{1}\right)\;i=1,2,\cdots ,s$$

where $r_{i}={\left[r_{11},r_{11},\cdots ,r_{1s}\right]}^{\prime }$ is for the i training set sample vector and $a_{i}=\left[a_{i}^{1},a_{i}^{2},\cdots ,a_{i}^{s}\right]$. Compared with the traditional radial basis function network, the generalized regression neural network uses the output matrix B of the training set as the connection weight value W between the hidden layer and the output layer.

④ Calculation of output layer node results. Upon determining the connection weight value between the hidden layer and the output layer, the output layer node results are obtained, as detailed in Equations (9) and (10).

(9) $${\mathbb{N}}_{i}=\frac{LW_{2,1}a_{i}}{\sum_{j=1}^{s}a_{i}^{j}}\;i=1,2,\cdots ,s$$

(10) $$O_{i}=\mathrm{purelin}\left({\mathbb{N}}_{i}\right)={\mathbb{N}}_{i}\;i=1,2,\cdots ,s$$

**Figure 3:**
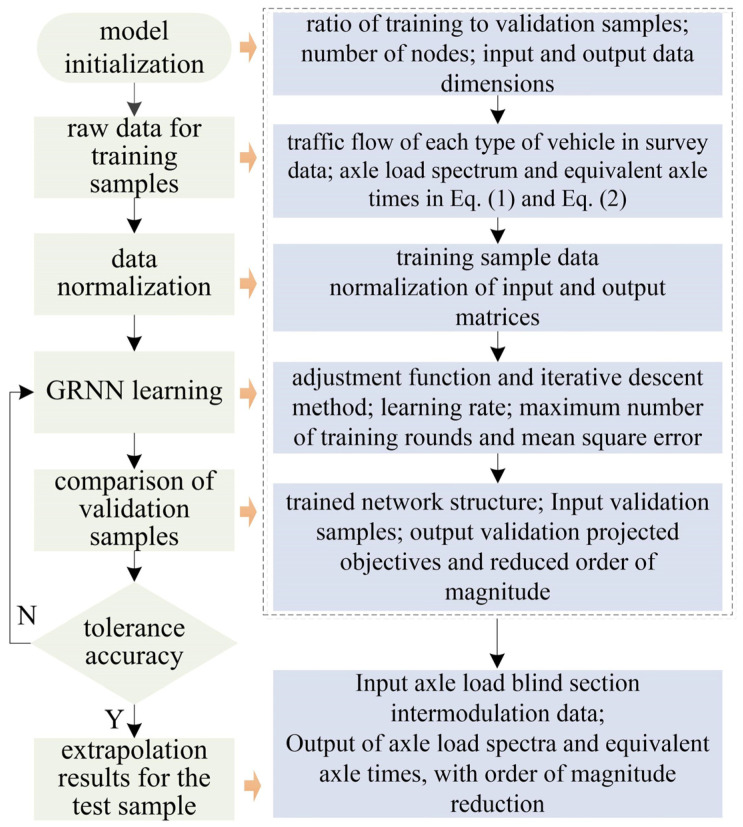
Implementation process of the GRNN model.

The specific flow steps of the GRNN model are outlined in Figure 3. For the sampled road sections, the model input consists of the corresponding flow of various vehicle types in the intermodulation data, while the output comprises the highway axle load spectrum and equivalent axle volumes. The sample data are partitioned into a training set and a verification set to facilitate error discrimination in the calculation results. Finally, a blind section of axle load detection, encompassing the intermodulation data, is utilized as the test set. The corresponding section’s output target value is computed using the GRNN. In this paper, we chose to use the Generalized Regression Neural Network (GRNN) over other deep learning models (such as LSTM and CNN) or simpler models (like linear regression) because GRNN excels in handling nonlinear relationships and limited sample data. Based on the Radial Basis Function (RBF) network, GRNN can converge quickly and achieve a global optimal solution, which is particularly important for calculating axle load spectra and equivalent axle volumes. We compared the GRNN model with traditional statistical methods and other machine learning models (such as SVM and RF). The results showed that GRNN performed better in terms of prediction accuracy and computational efficiency.

### 3.3. Data Description

The comprehensive data set utilized in this study integrates an extensive array of traffic observation and survey stations, along with meticulously collected axle load data detection points. This rich dataset is composed of continuous daily records meticulously gathered from an intricate network of highway sensors, ensuring a high degree of temporal resolution and accuracy. The dataset serves as a foundational resource for in-depth analysis and modeling of traffic patterns and infrastructural stress factors. The training set, designed to encapsulate a wide range of operational conditions, encompasses dynamic weighing stations, toll stations, and critical bridge health monitoring sections. These elements are strategically selected to represent the diverse and dynamic nature of highway traffic loads and structural responses. By incorporating such a broad spectrum of data sources, the training set is well-equipped to facilitate the development of robust and generalizable models capable of predicting complex traffic behaviors and structural stresses.

The verification set is specifically tailored to focus on axle load investigation station sections. This deliberate selection allows for a targeted assessment of the models’ ability to accurately predict axle loads, which are crucial for understanding pavement wear, bridge stability, and overall road infrastructure longevity. Both the training and verification sets include intermodulation data, which provides valuable insights into the interplay between different traffic components and their combined impact on road infrastructure. Furthermore, the inclusion of axle load data across these sets ensures a comprehensive evaluation of the models’ performance under varying loading conditions.

For missing values and outliers in sensor data, we employed interpolation and threshold methods. For missing values, we used the average of the surrounding data points for interpolation. For outliers, they were identified and removed based on preset threshold ranges and replaced with the average of adjacent normal values.

## 4. Case Studies

**Figure 4:**
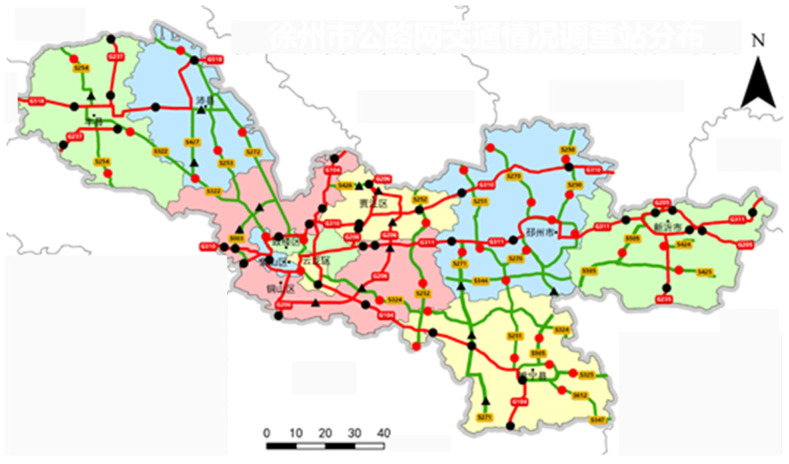
Traffic survey stations for the highway network in Xuzhou.

**Figure 5:**
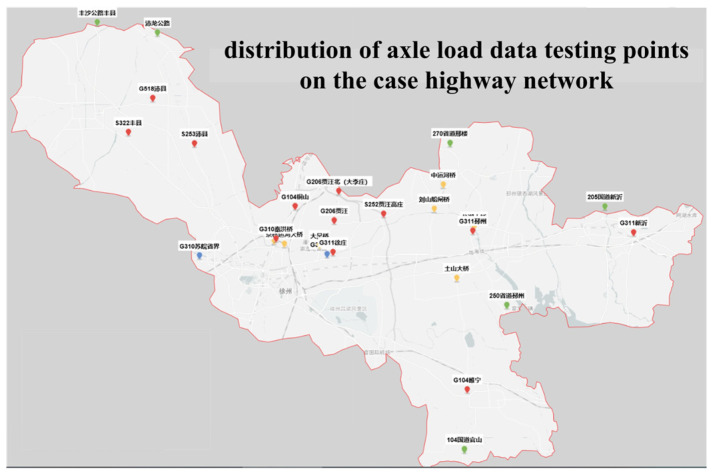
Axle load detections for the Xuzhou highway network.

A study was conducted on the highway network of Xuzhou City, Jiangsu Province. Figure 4 and Figure 5 illustrate the distributions of traffic observation and survey stations and axle load data detection points, respectively. The dataset comprised continuous daily records from 1 September to 30 September 2021. The training set encompassed dynamic weighing, toll station, and bridge health monitoring sections, while the verification set focused on axle load investigation station sections. Both sets included intermodulation and axle load data. The test set consisted of other road sections within the network, incorporating interchange data. The implementation environment comprised MATLAB R2019b and SQL Server 2017. Both charts and tables have corresponding supplementary explanations, providing more contextual information. The meaning behind the data is also analyzed to help readers better understand the research results.

**Figure 6:**
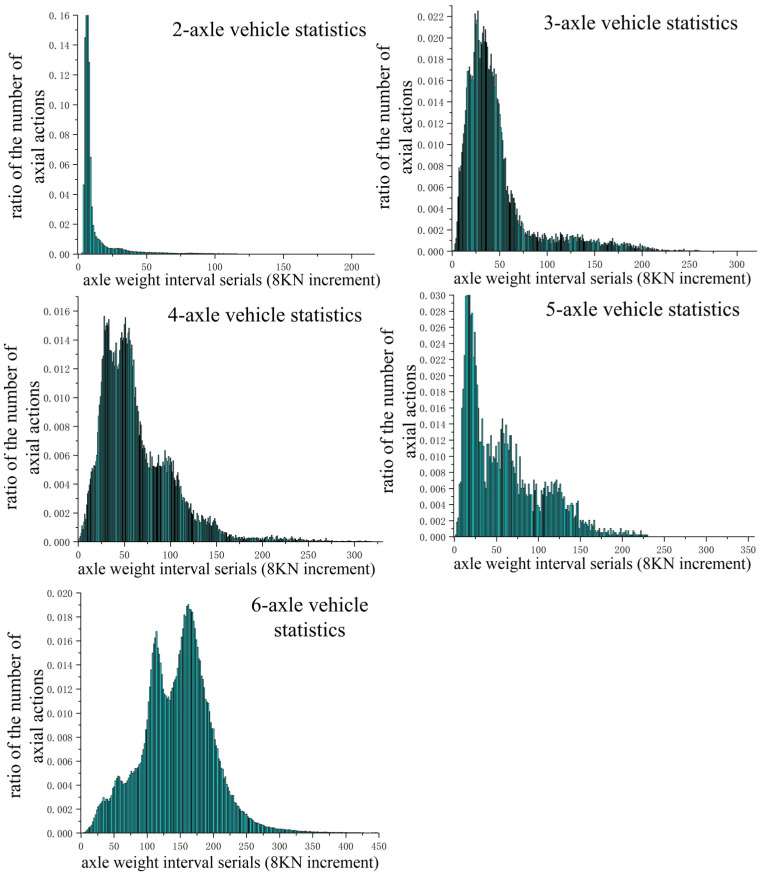
Average axle load spectrum of the Xuzhou highway network.

Figure 6 depicts the computed average axle load spectrum for the Xuzhou highway network. The overall distribution trend of the lower axle load spectrum for each vehicle model with varying axle counts aligns with actual conditions. Notably, 2-axle vehicles exhibit a pronounced “single-peak” distribution, typically not a focus in heavy-haul traffic. The 3-axle vehicles show a broader axle load span, with higher values on the right side of the “crest”, indicating more prevalent overloading. Among trucks, 4-axle vehicles are second to 6-axle vehicles in number, with a concentrated axle load distribution near the “left peak” and a relatively uniform distribution across Xuzhou highway sections. The 5-axle vehicles are the least numerous but exhibit a wide axle load span due to the presence of severely overloaded vehicles. The 6-axle vehicles dominate the truck population, with a significantly higher “right peak” amplitude compared to the left band, reflecting a greater number of fully loaded vehicles. This model is a primary focus in heavy-duty traffic. Additionally, the right side span of the 6-axle axle load spectrum’s “right peak” is narrow, indicating minimal severe overloading within the Xuzhou highway network.

The deviations among different vehicle types may be related to factors such as vehicle distribution, load characteristics, and sensor accuracy. To mitigate these deviations, we will further optimize the data preprocessing steps and consider introducing vehicle-specific correction factors. Table 2 presents the calculated results and comparisons of the average equivalent axle volumes for axle load detection sections in the Xuzhou highway network, corresponding to the detection points in Figure 5. The proposed deep learning method reveals distinct output target patterns across different models. Specifically, the calculated results for 2-axle, 5-axle, and 6-axle vehicles are smaller than the statistical values, whereas those for 3-axle and 4-axle vehicles are larger. This discrepancy arises from the scattered nature of 3-axle and 4-axle models, with a higher proportion of semi-trailers, weaker single-axle representation, and more challenging data normalization compared to 2-axle, 5-axle, and 6-axle vehicles. From an absolute deviation perspective, 6-axle vehicles exhibit the lowest calculation accuracy, primarily due to the inclusion of super-large trucks and container vehicles, whose axle load detections are subject to statistical hardware-induced variability.

**Figure 7:**
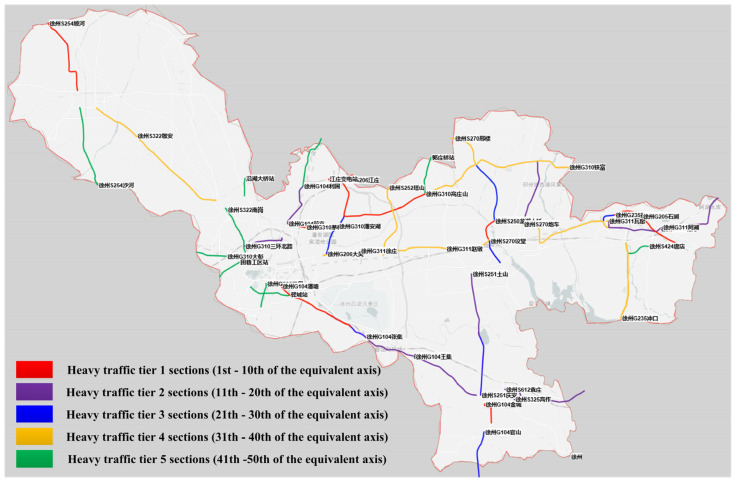
Distribution of heavy links of the Xuzhou highway network.

For road sections lacking axle load detection capabilities, four spatially distant sections were selected, including high-traffic (G206 Jiawangluzhuang, Tushan Bridge, Zhongyun) and low-traffic (S253 Pei County) sections. Using intersection data as input, the equivalent axle volumes were computed, with results displayed in Table 3. These results align with the overall pattern observed in Table 2. Furthermore, equivalent axle volumes were calculated for 331 sections of the Xuzhou highway network. Based on heavy-haul traffic classification rules [9], the top 50 sections (representing ≥15% of the network) were classified as heavy-haul road sections and divided into five levels, as shown in Figure 7. These sections and their grades serve as a foundation for the management department to determine highway maintenance funds and formulate maintenance plans within their jurisdiction. By applying the method proposed in this study, we accurately identified areas with frequent heavy vehicle traffic, guiding targeted maintenance strategies that effectively extended pavement life and reduced maintenance costs. Considering computational complexity, we will adopt a distributed computing architecture to process large-scale data from national or international highway networks. Additionally, we will develop a user-friendly interface to enable non-technical personnel to easily use our model.

## 5. Conclusions

This study proposes a data-driven approach based on a generalized regression neural network model for calculating axle load spectrum and equivalent axle times on highways and national/provincial roads, filling a gap in existing research. Secondly, through multi-source data fusion, this study enhances the coverage and accuracy of axle load data, providing more reliable data support for road maintenance decisions. Finally, validated with the case study of the Xuzhou highway network, the results demonstrate the good prediction accuracy and practicality of the proposed method, which can provide a scientific basis for the distribution and classification of heavily loaded road sections within the region, thereby guiding the allocation of road maintenance funds and the formulation of maintenance plans.

While this study is based on data from the Xuzhou highway network, we plan to apply the model to more regions and under different climatic conditions in future work to validate its broad applicability. Within the established generalized regression neural network model, the panel data characteristics among indistinguishable variables may induce early regression in deep learning scenarios with limited training samples. Future research could explore data dimensionality reduction techniques, such as incorporating a principal component analysis step, to pre-classify independent variables effectively.

## Figures and Tables

**Table 1:** Classification relationship of vehicle types.

Serial Number	Intermodulation Data Model	Axle Data Vehicle
1	Small and medium-sized passenger cars, large passenger cars, small trucks, medium trucks	2-axle car
2	Lorry	3-axle car
3	Large truck, extra-large truck, container truck	4-axle car
4	Extra-large truck, container truck	5-axle car
5	Extra-large truck, container truck	Car with 6 axles and above

**Table 2:** Estimation results of equivalent volumes of axle load detection links.

Vehicle Type	Statistical Equivalent Axes/Times	Calculated Equivalent Axle Times/Times	Deviation/%
2-axle car	15,859.34	13,046.11	−17.74
3-axle car	40,398.77	43,331.26	7.26
4-axle car	39,702.08	46,585.67	17.34
5-axle car	2074.57	1739.21	−16.17
6-axle car	277,730.29	219,559.46	−20.95
Total	375,765.07	333,412.99	−11.27

**Table 3:** Calculation examples for equivalent axle volumes of the blind links of axle load detection (unit: times).

Vehicle Type	G206 Jiawang Luzhuang	S253 Pei County	Tushan Bridge	Middle Canal
2-axle car	52,655.89	34,069.55	63,927.21	91,332.08
3-axle car	45,272.87	32,013.93	45,440.70	47,286.86
4-axle car	41,139.11	33,561.50	37,032.88	43,134.67
5-axle car	3165.89	2168.06	2025.55	2739.92
6-axle car	291,822.93	226,829.52	236,733.85	252,769.48
Total	434,056.69	328,642.56	385,160.18	437,263.01

## Data Availability

The raw data supporting the conclusions of this article will be made available by the authors on request.
